# The use of residual analysis to improve the error rate accuracy of machine translation

**DOI:** 10.1038/s41598-024-59524-3

**Published:** 2024-04-23

**Authors:** Ľubomír Benko, Dasa Munkova, Michal Munk, Lucia Benkova, Petr Hajek

**Affiliations:** 1 Constantine the Philosopher University in Nitra, Tr. A. Hlinku 1, 949 01 Nitra, Slovakia; 2 Science and Research Centre, University of Pardubice, Studentská 84, 532 10 Pardubice, Czech Republic

**Keywords:** Computer science, Computational science

## Abstract

The aim of the study is to compare two different approaches to machine translation—statistical and neural—using automatic MT metrics of error rate and residuals. We examined four available online MT systems (statistical Google Translate, neural Google Translate, and two European commission’s MT tools—statistical mt@ec and neural eTranslation) through their products (MT outputs). We propose using residual analysis to improve the accuracy of machine translation error rate. Residuals represent a new approach to comparing the quality of statistical and neural MT outputs. The study provides new insights into evaluating machine translation quality from English and German into Slovak through automatic error rate metrics. In the category of prediction and syntactic-semantic correlativeness, statistical MT showed a significantly higher error rate than neural MT. Conversely, in the category of lexical semantics, neural MT showed a significantly higher error rate than statistical MT. The results indicate that relying solely on the reference when determining MT quality is insufficient. However, when combined with residuals, it offers a more objective view of MT quality and facilitates the comparison of statistical MT and neural MT.

## Introduction

Although relying on human translation offers more accuracy and fluency, human translation is of limited efficiency and it is challenging for it to meet the needs of long text translation^1^. This limitation stimulates the search for new approaches to translation. One such approach is the implementation of intelligent algorithms within machine translation (MT) system. Various algorithms address the issues of MT system such as RNN encoding–decoding in existing log-linear SMT, transfer learning method, self-attention mechanism, unsupervised training algorithm, the adversarial augmentation method, reinforcement learning, neural MT (LSTM and transformer), hybrid (neural MT + statistical MT), rule-based MT, phrase-based MT, and others^2^. Currently, machine translation employs deep neural network (NN) learning, which initially learns rules and then automatically produces translations. This approach has yielded very good results for tasks with sufficiently labelled data for learning. However, if there is little tagged data, machine translation produces poor performance^3^. The primary obstacle for market-oriented neural MT systems or applications lies in its weak translation quality that fails to meet users’ needs^4^. MT evaluation is a fundamental step in improving the performance of MT systems. The continuous enhancement of the performance of current neural MT systems is closely tied to research on evaluating the quality of MT output based on sentence comparison^4^. This comparison involves two inseparable aspects—qualitative/human and quantitative/automatic evaluation. The first serves as the foundation and guiding principle for the second, while the latter represents the digital outcome of the former.

Two main approaches exist for evaluating MT systems—human/manual and automatic evaluation. Blur criteria and scales for manual translation quality, along with different human evaluator sensitivity to translation errors may result in the judge subjectivity, which can be reflected in the poor consistency and instability of the evaluation results^5^. Human evaluation is an effective way to assess translation quality, but is challenging to find reliable bilingual annotators^6^. In addition to poor consistency and subjectivity, manual evaluation is both financially and time-consuming; however, unlike automatic evaluation, it does not require a reference translation. The advantages of automatic evaluation lie in its objectivity, consistency, stability, speed, reusability, and language independence. It is cost-effective and easy to use for comparing multiply systems, but at the expense of quality^6^. Furthermore, automatic evaluation requires reference—human translation (gold standard)—since the evaluation is based on comparing MT output with reference translation. Automatic MT evaluation metrics compare overlapping words between MT output and reference (e.g.^7–9^ and others). Automatic metrics of MT evaluation only capture lexical similarity and correctly measure neither semantic and grammatical diversity, nor syntactic structures^6,10^.

The automatic evaluation metrics applied in MT evaluation can be divided into untrained—lexical metrics and trained—supervised and unsupervised metrics^6^. The untrained metrics measure lexical similarity/distance (overlap with a reference) using a mathematical formula or heuristic methods at the word-level (N-gram-based metrics such as BLEU, METEOR or NIST; or edit distance-based metrics such as TER or WER) or at the character-level (such as characTER). Within the trained metrics, we can distinguish embedding-based metrics from supervised metrics. The embedding-based metric measures lexical similarity using machine learning techniques or deep learning algorithms (automatic metrics such as MEANT or BEER). The supervised metric trains regression models using labelled data, annotated by humans such as the COMET metric^6^.

The BLEU (Bilingual Evaluation Understudy) evaluation index is the standard method used in the automatic evaluation of the quality of the MT system through its product. The BLEU index is calculated through three components that require minimal human intervention: (1) n-gram-based precision of the MT output and the reference, (2) brevity penalty to prevent overfitting of sentence length, and (3) clipping for calibration of continuous word appearance^6^. The BLEU index ranges from 0 to 1, with a higher value indicating more accurate matching (overlap) with the reference translation. On the other hand, the closer to zero, the more machine translation deviates (differs) from reference translation^11^. However, this index does not reflect the degree of this deviation and its gravity. Its poor performance at sentence-level and inadequate handling of recall are its main limitations^12^.

Moreover, many studies have shown that BLEU has a low correlation with human evaluation, especially in cases of inflectional languages, which has led to the development of various BLEU variants^13–15^. Additionally, BLEU is not always reported consistently due to divergences in the tokenization and normalization schemes used^16^.

The BLEU index only determines accuracy with the reference (similarity), which is neither helpful in improving nor in optimizing the MT system. To know or understand what needs to be improved, we need to know the error rate. For this reason, in our paper, we focused on the error rate (edit distance) and not on the lexical similarities (accuracy) of MT systems.

Word error rate (WER) measures Levenshtein edit distance, i.e., it computes the minimum edit distance to transform a MT output into a reference through edit operations such as insertions, substitutions, and deletions of words necessary to transform one string into another without allowing the words reordering^17^:

$$WER = \frac{{\# {\text{ of insertions}} + deletions + substitutions}}{reference\, lenght}.$$

The limitation of the WER metric lies in penalizing word changes within a sentence. Several variants overcome this limitation, such as position-independent word error rate metric^18^ or translation edit rate metric^8^.

Position-independent word error rate (PER) is based on the WER metric but ignores the word order in both MT output and reference translation^18^.

Translation edit rate (TER) is defined as the minimum number of edit operations, including shift (a moving action or block movement), required to change a MT output to an exact match with a reference^8^:

$$TER = \frac{{\# {\text{ of insertions}} + deletions + substitutions + shifts}}{reference\, lenght}.$$

In comparison with WER, which focuses on word operations only, TER considers shifts as part of edit operations. The higher the score of the error rate metrics, the worse the translation quality, and vice versa. The main motivation for using character-based metrics is their improved performance in evaluating morphologically rich languages like Slovak or other Slavic languages^19,20^.

CharacTER is a character-level metric inspired by the TER metric^21^. It is defined as the minimum number of character edit operations required to match a MT output with a reference, normalized by the length of the MT output:

$$CharacTER = \frac{{\# {\text{ of insertions}} + deletions + substitutions + shifts}}{MT\, output\, lenght}.$$

CharacTER first performs shift edits at the word-level; then, the shifted MT output sequence and the reference are split into characters, and the Levenshtein distance between them is computed.

Cross-lingual optimized metric for the evaluation of translation (COMET) is a PyTorch-based framework for training highly multilingual and adaptable MT evaluation models that can function as metrics^22^. It supports both architectures: the estimator model (trained to regress directly on a quality score) and the translation ranking model (trained to minimize the distance between “good” MT output and its corresponding reference/original source).

The most commonly used approach to determine the ability of automatic metrics to substitute human evaluation metrics is to compare the correlations between human evaluation metrics and the scores of automatic metrics^6^. However, it is still only a score (a number from the < 0, 1 > interval) that does not indicate the level of translation error rate at the segment/sentence/text level within the corpus. Additionally, automated metrics provide varying results and varying degrees of correlation with human evaluations, which are often inconsistent themselves. The translation quality of a pair of MT systems often relies on the differences between automatic scores (BLEU index) to draw conclusions without performing any further assessment^23,24^.

This motivated us to search for other techniques that would be suitable for comparing translation quality and help us to identify segments/sentences/texts within a corpus that vary extremely (significantly) in translation quality, but with minimal human intervention. The advantage of using residuals when comparing translations is the ability to detect specific segments/sentences/texts within the corpus that deviate significantly from the golden-standard translation.

Residual analysis and error analysis are closely related analyses; both measure a distance (deviation or error). Residual analysis evaluates a regression model's validity by examining the differences between observed values and predicted values by the model; in our case, the model is the MT model.

The deviation or error is the distance of the observed value from the predicted/expected value, i.e., residuals represent the distance of observed values from predicated values:

$$Residual\, value_{i} = Observed\, value_{i} - Predicted\, value_{i} , i = 1,2, \ldots ,N,$$

where, in our research, *N* represents the number of examined texts in the data set, observed value is represented by the neural MT error rate and the predicted/expected value by the statistical MT error rate of a given text.

Extreme distances between the examined models (MT systems in our research) are identified based on the $\pm 2sigma$ rule, similar to outliers in residual analysis:

$$Mean\left( {Residual\, value_{i} } \right) \pm StdDev\left( {Residual\, value_{i} } \right), i = 1,2, \ldots ,N,$$

where the residual values represent the differences in the error rate of the examined MT models, neural MT system and statistical MT system in our case.

Residuals allow us to identify patterns, better understand and interpret model errors, and subsequently eliminate, correct, or analyze them, as well as their influence on MT quality^25^.The aim of the study is to compare two different approaches to machine translation—statistical and neural—using automatic MT metrics of error rate and residuals. We examined four available online MT systems (statistical Google Translate, neural Google Translate, and two European commission’s MT tools—statistical mt@ec and neural eTranslation) through their products (MT outputs).

The statistical MT (SMT) systems are represented by Google Translate (GT_SMT)^26^ and mt@ec (the European Commission’s MT tool)^27^ and their transformations into neural MT (NMT) systems, which are represented by Google Translate (GT_NMT)^28^ and eTranslation (the European Commission MT tool)^29^. The shift from mt@ec to eTranslation improved the translation quality, speed, and security of the interface. Google team made the same transformation in September 2016; it switched to Google neural machine translation, focusing on an end-to-end learning framework that learns from millions of examples and provides significant improvements in translation quality^30^.

The main objective consists of three partial objectives:

- The first objective lies in the comparison of statistical MT systems and neural MT systems based on the automatic MT metrics of error rate (WER, PER, and TER).
- The second objective aims to identify or detect machine-translated segments/sentences/texts that deviate significantly from human translations based on the score of error rate metrics and residuals. This includes identifying texts in which statistical MT was closer to human translation than neural MT or vice versa.
- The third objective involves verifying the validity of the obtained results through metrics such as BLEU and COMET, as well as the characTER metric, which correlates better with human evaluation in the case of morphologically richer languages^19,20^.

The translation directions were from English and German into an inflectional and a low-resourced Slovak. Moreover, Slovak belongs to one of the official languages of the European Union.

The structure of the paper is as follows. The second section contains related work in the field of automated MT evaluation and a comparison of various MT systems. The third section describes the used data set and the applied research methodology. The subsequent section focuses on the research results based on the evaluation of error rate metrics and residuals. The fifth section offers a discussion of the results. The last section comprises research conclusions.

## Related work

Statistical MT and neural MT are the most extensively used architectures within the MT systems^31^.

Pinnis et al.^32^ compared the NMT and phrase-based SMT systems for highly inflected and low-resourced languages. They compared large and small bilingual corpora, focusing on six language pairs: [Latvian, Estonian]-English, Estonian-Russian, and vice versa. MT evaluation was conducted using the automatic evaluation metrics (BLEU, NIST, and ChrF2) and manual error analysis. The error analysis was focused on identification morphological, syntactical, and lexical errors. The results showed that the NMT system produced twice as many errors in lexical choice (wrong or incorrect lexical choice) as the phrase-based SMT system. On the other hand, the NMT system demonstrated much better grammatical accuracy (forms and structure of words, and word order) than the SMT system.

Yang et al.^33^ examined translation quality from ancient Chinese to modern Chinese. They proposed a novel automatic evaluation model—dual-based translation evaluation, without multiple references. To compare the results, BLEU and the Levenshtein distance were used as baselines. They proved that dual-based translation evaluation achieved better agreement, and/or concordance with human evaluation (human judgements).

Fomicheva and Specia^34^ conducted a broad meta-evaluation study of automatic evaluation metrics. They evaluated more than 20 automatic evaluation metrics on multiple data sets (WMT16 data set, MTSummit17 English–Latvian data set, Multiple-Translation Chinese data set, WMT17 Quality Estimation German–English data set, GALE Arabic–English data set, and EAMT11 French–English data set). Data sets also contained manual assessments based on different quality criteria (adequacy, fluency, or PE effort) collected using several different methods. The meta-evaluation was conducted based on three aspects: MT quality, MT system types, and manual evaluation type. They showed that the accuracy of automatic MT evaluation varies depending on the overall MT quality. They showed that the automatic metrics perform poorly when faced with low-quality translations, but additionally that evaluating low-quality translations is also more challenging for humans. They also showed that metrics are more reliable when evaluating neural MT than statistical MT systems. Metric performance can be affected by various factors, such as text-domain, language-pair, or type of MT system.

Moghe et al.^35^ evaluated nine metrics consisting of string overlap metrics, embedding-based metrics, and metrics trained using scores from human MT evaluation on three extrinsic tasks (dialogue state tracking, question answering, and semantic parsing) covering 39 unique language pairs. They showed that interpreting the quality of the produced MT translation based on a number is unreliable and difficult. They also showed that scores provided by neural metrics (e.g. COMET) are not interpretable, in large part due to having undefined ranges, and also that it is unclear if automatic metrics can reliably distinguish good translations from bad at the sentence level.

Alvarez-Vidal and Olivier^23^ found that automatic metrics such as BLEU were intended to be used as a development tool and cannot be blindly used to assess MT systems without taking into account the final use of the translated text. They recommend a two-step MT evaluation which can ensure the quality of the MT output. They compared two different NMT engines—the commercial online available DeepL NMT system and a system trained on news-domain by the authors for the English–Spanish language pair. They showed that automatic metrics used (BLEU, NIST, WER, TER, EdDist, and COMET) yield better results for the NMT system trained by the authors, except for COMET.

Almahasees^36^ compared the MT outputs of Google Translate and Microsoft Bing Translator (both based on SMT). The used data contained political news in English and were translated into Arabic. The data were evaluated using the automatic evaluation metrics, and the results showed better results for MT outputs produced by Google Translate. Later, Almahasees^37^ conducted similar research with journalistic texts for the language pair English-Arabic, but with MT systems operating on neural networks. He compared the MT outputs based on automatic MT evaluation metrics of error rate. The results showed similar results for both MT systems in orthography and grammatical accuracy. The difference was found in the case of lexis, where the neural MT (Google Translate) achieved better results than the statistical MT (Bing).

Marzouk and Hansen-Schirra^38^ focused on controlled languages (CLs) to improve the quality of NMT output. They compare the impact of applying nine CL rules on the quality of MT output produced by five MT systems (Google, Bing, Lucy, SDL, and Systran, i.e., neural, rule-based, statistical, and two hybrid MT systems) by applying three methods: error annotation, human evaluation, and automatic evaluation (TERbase and hLEPOR). The data set consisted of 216 source sentences of technical-domain translated from German into English. They showed that the NMT does not require CL rules; i.e., before and after applying the CL rules, NMT system showed the lowest number of errors.

Li and Wang^39^ focused on the optimization of automatic MT evaluation. They applied representative ‘list-wise learning to rank’ approach, ListMLE. The selection of features was motivated by the BLEU-n metrics, phrase-based SMT, and NMT. They used the data sets released for WMT'2014 and WMT'2015 metrics tasks. To evaluate the results of the experiment, they compared the list-wise approach with the most used metrics, such as BLEU-n, METEOR, TER, etc. The results showed that the novel approach achieved better results than the above-mentioned metrics.

Singh and Singh^40^ focused on MT quality for low-resource languages. They aimed at an NMT system that should improve the translation quality for the English-Manipuri language pair. They compared multiple approaches such as SMT, RNN, and transformer architecture. The results showed a higher quality translation in terms of statistically significant automatic scores and manual evaluation compared to the statistical and neural supervised baselines, as well as the pretrained mBART and existing semi-supervised models.

Shterionov et al.^41^ compared phrase-based SMT and NMT systems based on lexical similarities. They applied automatic evaluation metrics (BLEU, TER, and F-measure) to assess the performance of MT systems. Based on the same data set, they built five NMT and phrase-based SMT engines for various language pairs. They showed that the quality evaluation scores indicated better performance for the PBSMT engines, contrary to human evaluation. They suggested that automatic evaluation metrics (BLEU, TER, and F-measure) are not always convenient for evaluation and do not correspond with NMT quality.

Tryhubyshyn et al.^42^ examined the relationship between MT system quality and QE system performance. They showed that QE systems trained on lower-quality MT translations (a mix of translations from different MT models) tended to perform better than those trained on higher-quality MT outputs (translations from one MT system).

As mentioned in the introduction, automated metrics (such as BLEU) yield varying results depending on the reference translation, text domain and languages. They draw conclusions without performing further evaluation or analysis, such as error analysis. Moreover, when the results of automatic evaluation were compared with those of manual evaluation, their correlation reached different degrees of agreement. Additionally, evaluators in the manual evaluations were often inconsistent regarding the error rate of the machine translation. These findings are also supported by the studies focused on Slavic languages or low-resource languages.

The aforementioned studies (as well as ours in the first objective) found that, on average, NMT is better than SMT. However, our proposed approach through residual analysis (regardless of which automatic metric is used) identifies segments that, on the contrary, show higher SMT quality. We have shown that our approach is suitable not only for automatic metrics of accuracy, but also for automatic metrics of error rate, which distinguishes us from all previous studies focused on Slovak so far. Moreover, it turns out that it is more appropriate not to consider the raw score of automatic metrics within the MT quality evaluation and comparison of NMT and SMT, but their distance (difference). Analyzing differences will enable us to evaluate the MT quality of individual segments.

## Materials and methods

This study focuses on comparing NMT systems (represented by Google Translate and eTranslation) and SMT systems (represented by Google Translate and mt@ec, the European Commission’s MT tool, which has later transformed into neural MT- eTranslation).

The statistical machine translated articles were obtained in 2016 from both, Google Translate (GT_SMT) and the European Commission’s DGT tool (mt@ec). Later, in 2021, the same articles were machine-translated by the NMT engines Google Translate (GT_NMT) and the European Commission’s DGT tool (eTranslation).The translation directions were from English and German into Slovak, where Slovak is a synthetic language containing inflected morphology and with loose word order^43^. Human translation and post-editing of machine translation were conducted in interactive online system OSTEPERE^25,44–47^.

The examined articles were tokenized and aligned using the Hunalign tool^48^ in the following order: source sentence with one human translation (HT), four machine translations (MTs), and one post-edited machine translation (PEMT).

The evaluation of the two different MT systems was conducted through automatic metrics of error rate (WER, PER, and TER). We aimed to identify the errors produced by the examined MT systems and determine whether changing the architecture of the MT systems resulted in decreasing to produce the same errors or, on the contrary, whether they start to create new ones. To verify the validity of the obtained results of the error rate, we used the metrics of accuracy—BLEU and COMET, and also character-based metric of error rate—characTER.

### Dataset

The data set comprises articles published by the British online newspaper The Guardian and the German online newspaper Der Spiegel, along with their machine and human translations. The corpus consists of eight data sets, and/or two English-Slovak and German-Slovak corpora: (1) articles written in English and German as source texts, (2) articles machine-translated from English and German into Slovak by four different MT engines (by SMT in 2016 and by NMT 2021), (3) human-translated articles from English and German into Slovak by professional translators (both in 2016), and (4) post-edited machine-translated articles by professional translators (in 2016).

The lexico-grammatical structure of the dataset^49^ was obtained using Stanza^50^, an automatic morphological annotator tool (Table 1).

**Table 1:** Dataset composition of (a) English MT outputs/HT and (b) German MT outputs/HT.

Feature name	GT_SMT	GT_NMT	mt@ec_SMT	eTransla-tion_NMT	Human translation
(a) Feature type					
Readability					
Average sentence length (words)	19.39	19.12	17.91	19.09	19.84
Average word length (characters)	5.43	5.59	5.75	5.55	5.69
Number of short sentences (n < 10)	18.13%	18.75%	21.88%	18.13%	15.63%
Number of long sentences (n ≥ 10)	81.88%	81.25%	78.13%	81.88%	84.38%
Lexico-grammatical					
Frequency of nouns	22.65%	22.06%	23.80%	21.90%	21.99%
Frequency of adjectives	10.84%	10.96%	11.81%	10.85%	10.59%
Frequency of verbs	9.30%	9.88%	8.79%	9.74%	10.78%
Frequency of determiners	4.44%	4.48%	4.52%	4.41%	5.17%
Frequency of adpositions	9.63%	9.68%	9.42%	9.46%	9.35%
Frequency of proper nouns	4.77%	4.45%	4.67%	4.88%	4.23%
Frequency of coordinating conjunctions	3.31%	3.14%	3.56%	3.26%	3.40%
Frequency of subordinating conjunctions	3.34%	3.53%	2.69%	3.65%	2.75%
Frequency of interjections	0.17%	0.17%	0.03%	0.11%	0.13%
Frequency of adverbs	3.50%	3.37%	3.29%	3.46%	3.56%
Frequency of pronouns	2.48%	3.26%	2.09%	3.21%	3.99%
Frequency of auxiliaries	4.42%	3.51%	3.95%	3.99%	2.94%
Frequency of numerals	3.75%	3.90%	4.16%	3.77%	3.61%
Frequency of particles	1.46%	1.70%	1.53%	1.59%	1.97%
Frequency of punctuations	14.62%	15.14%	14.44%	14.92%	14.42%
Frequency of others	1.30%	0.78%	1.26%	0.81%	1.13%
(b) Feature type					
Readability					
Average sentence length (words)	14.22	14.20	12.81	13.74	14.63
Average word length (characters)	5.44	5.54	5.90	5.60	5.65
Number of short sentences (n < 10)	36.14%	36.49%	41.86%	37.39%	33.09%
Number of long sentences (n ≥ 10)	63.86%	63.51%	58.14%	62.61%	66.91%
Lexico-grammatical					
Frequency of nouns	22.00%	22.88%	25.22%	22.79%	23.02%
Frequency of adjectives	9.98%	10.36%	10.61%	10.43%	10.53%
Frequency of verbs	9.11%	9.74%	7.94%	9.55%	9.79%
Frequency of determiners	4.23%	4.50%	3.59%	4.37%	4.57%
Frequency of adpositions	9.38%	9.74%	9.33%	9.80%	9.57%
Frequency of proper nouns	5.50%	5.40%	4.76%	5.59%	5.24%
Frequency of coordinating conjunctions	3.14%	2.90%	2.95%	2.84%	3.00%
Frequency of subordinating conjunctions	2.90%	2.63%	2.57%	2.78%	2.50%
Frequency of interjections	0.06%	0.04%	0.01%	0.03%	0.01%
Frequency of adverbs	4.83%	4.36%	3.95%	3.91%	4.47%
Frequency of pronouns	2.28%	2.95%	2.03%	2.84%	3.32%
Frequency of auxiliaries	3.68%	3.04%	3.33%	3.25%	2.72%
Frequency of numerals	3.09%	3.05%	3.27%	3.24%	2.97%
Frequency of particles	2.68%	2.70%	2.92%	2.61%	3.69%
Frequency of punctuations	16.04%	14.99%	16.42%	15.28%	13.85%
Frequency of others	1.11%	0.72%	1.09%	0.69%	0.74%

Due to the fact that the created corpora are composed of articles with the features of newspaper writing (own register), the examined corpora mainly consist of nouns, followed by verbs and adjectives. Regarding the readability of the examined translations (from EN to SK and also from DE to SK), there are unequal proportions of short (*n* < 10) and long (*n* >  = 10) sentences among MTs. The reduction in words within the sentence occurs frequently in statistical MT (mt@ec), which indicates word omission and a shift in meaning, and/or a certain loss of meaning (e.g., short sentences (*n* < 10) for EN: GT_SMT = 18.13%; GT_NMT = 18.75%; mt@ec_SMT = 21.88%; eTranslation_NMT = 15.63%; and for DE: GT_SMT = 36.14%; GT_NMT = 36.49%; mt@ec_SMT = 41.86%; eTranslation_NMT = 37.39%).

The readability results are also confirmed by corpus statistics (Table 1), where only adjectives are approximately equally distributed in all four MT outputs in both language directions (e.g., adjectives for EN: GT_SMT = 10.84%; GT_NMT = 10.96%; mt@ec_SMT = 11.81%; eTranslation_NMT = 10.85%; and for DE: GT_SMT = 9.98%; GT_NMT = 10.36%; mt@ec_SMT = 10.61%; eTranslation_NMT = 10.43%), compared to verbs or nouns (Table 1). This motivated us to investigate the differences between individual MT outputs, whether these differences are statistically significant and whether these differences cause grammatical or lexical errors in translation.

### Applied Methodology

**Figure 1:**
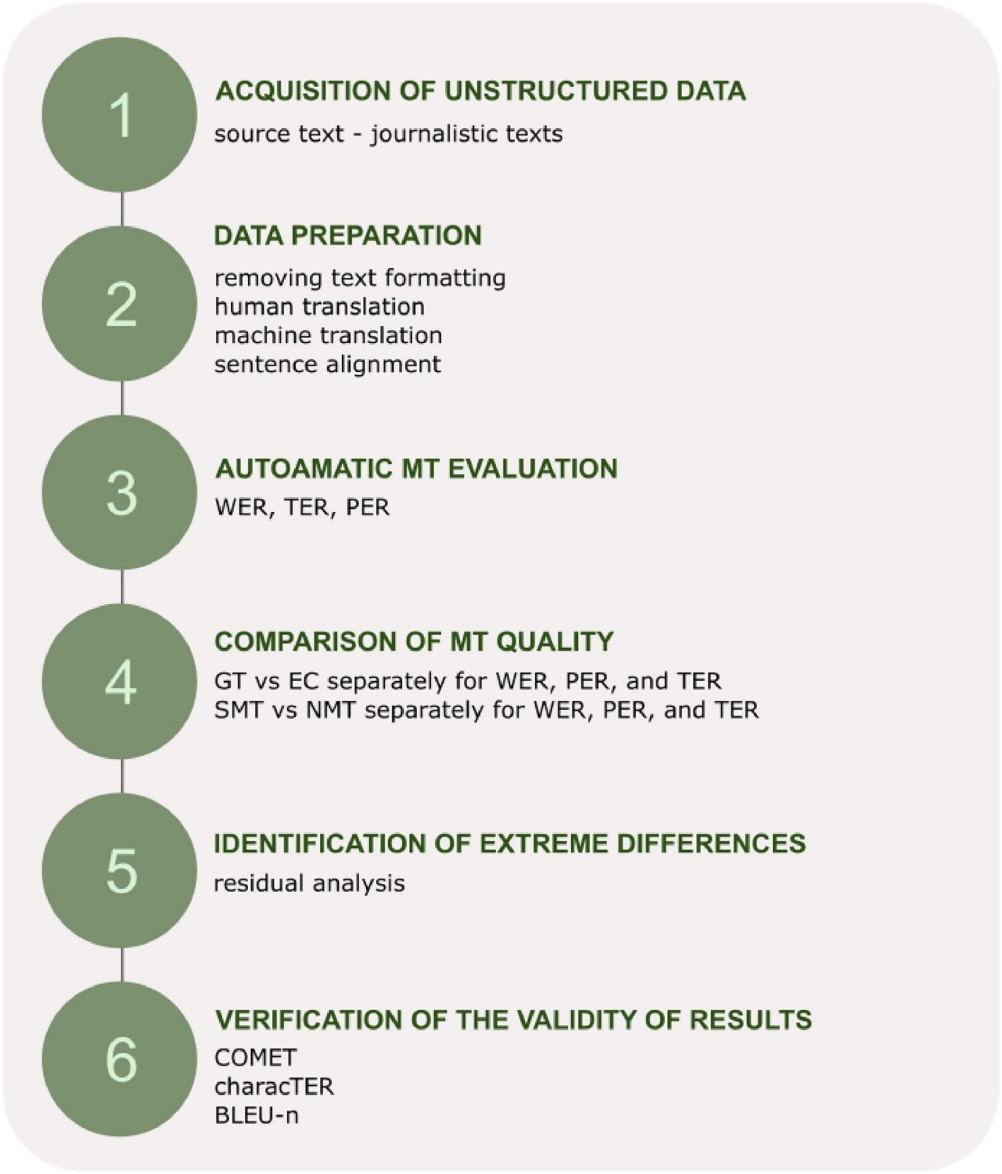
Methodology workflow diagram.

The applied methodology, inspired by other studies^51–53^, consists of these stages (Fig. 1):

1. *Acquisition of unstructured textual data* source text (journalistic texts). We focused on journalistic texts (newspaper writing) as they belong to the most read and translated texts by people. We chose the two most popular journals, from which we obtained all freely available texts from various fields (politics, sports, show business, and technology) published in the given year 2016.
2. *Data preparation* consisting of following tasks:
  - *Text pre-processing* removing text formatting, which can influence the MT quality (images or tables can divide the text inappropriately and produce bad translation).
  - *Human translation* the translation process was realized in the tailored system OSTEPERE, which offers user-friendly interface for human translators and post-editors. The system saved the human translations and post-edited machine translations into a database for further processing.
  - *Machine translation* automatic translation of the source text by MT engines (Google Translate [SMT | NMT], mt@ec [SMT], and eTranslation [NMT].
  - *Sentence alignment* the generated MT outputs and human translations are aligned with the source texts using Hunalign tool (based on the 1-to-1 principle).
3. Automatic MT evaluation using automatic metrics of error rate at the segment level. We applied automatic MT metrics based on the Levenshtein distance, which computes the minimum edit distance to transform a MT output into a reference through edit operations (insertions, substitutions, deletions, and shift of words necessary to transform one string into another). $WER\left( {h,r} \right) = \frac{{min\# \left( {I + D + S} \right)}}{\left| r \right|}$, where *r* is a reference of a hypothesis/MT output *h*, *I*—insertion, *D*—deletion, and *S*—substitution.
  The minimum number of edit operations is divided by the number of words in the reference^54^. $PER\left( {h,r} \right) = 1 - \frac{{n - {\text{max}}\left( {0, \left| h \right| - \left| r \right|} \right)}}{\left| r \right|}$, where *r* is a reference of a hypothesis/MT output *h*, *n* is the number of similar words^18^.
  $TER \left( {h,r} \right) = \frac{{min\# \left( {I + D + S + shift} \right)}}{\left| r \right|}$, where *r* is a reference of a hypothesis/MT output *h*, *I*—insertion, *D*—deletion, *S* – substitution, and *shift* (a number of changes in word order). Compared to WER, TER considers shifts as a part of edit operations. TER deals with more edit operations, allowing it to capture various differences in word order.
  The higher the score of error rate metrics, the worse the translation quality, and vice versa.
4. Comparison of MT quality based on (1) MT system used (Google Translate or European Commission’s DGT system) and (2) artificial intelligence approach to MT (statistical approach to MT or neural approach to MT).
  (i) We test the differences in the score of automatic MT metrics between two MT systems (Google Translate (GT) and the European Commission’s MT tool (EC)), separately for WER, PER, and TER.
  (ii) We test the differences in the score of automatic MT metrics between artificial intelligence approached to MT (statistical vs neural), separately for WER, PER, and TER.
    To test the differences between dependent samples (WER/PER/TER: EC_SMT, GT_SMT, EC_NMT, and GT_NMT), we use adjusted univariate tests for repeated measure due to the failure of the sphericity assumption (Mauchley sphericity test – WER: *W* = 0.849, *Chi-Square* = 25.886, *df* = 5, *p* < 0.001; PER: *W* = 0.916, *Chi-Square* = 13.795, *df* = 5, *p* = 0.017; TER: *W* = 0.846, *Chi-Square* = 26.336, *df* = 5, *p* < 0.001).
5. Identification of extreme differences between statistical and neural MT. To identify extreme values, we apply the residual analysis, i.e., $\left( {residual\,value} \right)_{i} = \left( {WER/PER/TER\, score\,of\,NMT\,text} \right)_{i} - \left( {WER/PER/TER\, score\,of{\text{ S\,}}MT\,text} \right)_{i}$ $i = 1,2, \ldots ,N$, where *N* is the number of examined texts in the dataset.
6. Validation of the obtained results –using automatic metrics BLEU, COMET, and characTER.
  We used one of the main models of COMET: *wmt22-comet-da*. This model uses a reference-based regression approach and has been trained on direct assessments from WMT17 to WMT20. It provides scores ranging from 0 to 1, where 1 represents a perfect translation. $CharacTER \left( {h,r} \right) = \frac{{min\# \left( {I + D + S + shift} \right)}}{{\left| {h\left( {characters} \right)} \right|}}$, where *h* is a hypothesis/MT output, *I*—insertion, *D*—deletion, *S* – substitution, and *shift*.

*BLEU-n*^7^ is a geometric mean of *n-gram precisions* with a *brevity penalty* (BP), i.e. penalty to prevent very short sentences:

$$BLEU{ }\left( n \right) = exp\mathop \sum \limits_{n = 1}^{N} w_{n} \log p_{n} \times BP,$$

where $w_{n}$ is weights for different $p_{n}$,

$$BP = \left\{ {\begin{array}{*{20}c} { 1, \quad if\, h > r} \\ {e^{{1 - \frac{r}{h}}} , \quad if\, h \le r} \\ \end{array} } \right. ,$$

where *r* is a reference of a hypothesis h.

## Results

### Automatic MT evaluation based on metrics of error rate

For all automatic metrics (WER, PER, and TER), the Mauchley sphericity test is significant (*p* < 0.05), i.e., the assumption is violated. We adjusted the degrees of freedom using the Greenhouse–Geisser adjustment. Based on the results of adjusted univariate tests for repeated measure (Greenhouse–Geisser adjustment) among GT_SMT, GT_NMT, mt@ec_SMT, and eTranslation_NMT, there are significant differences in MT quality concerning the scores of metrics of error rate (WER, PER, and TER: *G-G Epsilon* < 0.944, *G-G Adj. p* < 0.001). NMTs were of statistically significantly better quality than SMTs regardless of which MT tool (GT or the European Commission’s MT tool) was used. NMTs were lexically more similar to the references than SMTs.

Based on multiple comparisons (Table 2), there are significant differences in the score of metric WER between NMT (GT) and the others, as well as between NMT (eTranslation_NMT) and the others, but there is no difference between SMT (GT) and SMT (EC). Were identified three homogeneous groups (****p > 0.05) in terms of the agreement/concordance of the examined texts. NMT produced by GT achieved the lowest error rate (0.679) compared to other MTs. On the other hand, SMT produced by mt@ec achieved the highest error rate (0.800), but is very close to SMT produced by GT (0.778).

**Table 2:** Bonferroni (adjustment) post-hoc test for multiple comparisons of the metric WER between different MT systems (GT tools or EC tools) and approaches (statistical or neural) in the English–Slovak language pair.

English = 1	Mean	1	2	3
WER_GT_NMT	0.679		****	
WER_EC_NMT	0.715			****
WER_GT_SMT	0.778	****		
WER_EC_SMT	0.800	****		

****Homogenous groups *p* > 0.05.

In terms of lexical similarity, regardless of word order (PER metric), there is a difference between SMT, produced by GT tool or EC tool and neural MT, but there is no difference between neural MT produced by GT tool and EC tool (Table 3). Based on multiple comparisons (Table 3), were identified three homogeneous groups (****p > 0.05) in terms of the agreement/text similarity of the examined texts. Moreover, three out of four MTs achieved lower PER scores of error rate (*PER* ≤ 0.642) than all MTs evaluated by metric WER (*WER* ≥ 0.679).

**Table 3:** Bonferroni (adjustment) post-hoc test for multiple comparisons of the metric PER between different MT systems (GT tools or EC tools) and approaches (statistical or neural) in the English–Slovak language pair.

English = 1	Mean	1	2	3
PER_GT_NMT	0.548	****		
PER_EC_NMT	0.575	****		
PER_GT_SMT	0.642		****	
PER_EC_SMT	0.683			****

****Homogenous groups *p* > 0.05.

The TER values copy the WER values (Tables 2, 4). Based on multiple comparisons (Table 4), were identified three homogeneous groups (****p > 0.05) in terms of the agreement/text similarity of the examined texts. There are significant differences in the score of the metric TER between GT_NMT (neural GT) and the others, as well as between EC_NMT (eTranslation_NMT) and the others (Table 4), but there is no difference between GT_SMT (statistical GT) and EC_SMT (mt@ec_SMT). Neural MT produced by GT achieved the lowest error rate (0.674) compared to other MTs. On the other hand, statistical MT produced by mt@ec achieved the highest error rate (0.796), but is very close to statistical MT produced by GT (0.774).

**Table 4:** Bonferroni (adjustment) post-hoc test for multiple comparisons of TER metrics between different MT systems (GT tools or EC tools) and approaches (statistical or neural) in the English–Slovak language pair.

English = 1	Mean	1	2	3
TER_GT_NMT	0.674		****	
TER_EC_NMT	0.711			****
TER_GT_SMT	0.774	****		
TER_EC_SMT	0.796	****		

****Homogenous groups *p* > 0.05.

We applied the same analysis to machine-translated texts from German into Slovak. Due to the violation of the assumption of sphericity of the covariance matrix, we used modified tests for repeated measurements (Greenhouse–Geisser adjustment) to test the differences in MT quality among GT_SMT, GT_NMT, mt@ec_SMT, and eTranslation_NMT represented by the metrics of error rate (PER: *W* = 0.868, *Chi-sqr.* = 78.816, *df* = 5, *p* < 0.001; WER: *W* = 0.873, *Chi-sqr.* = 75.826, *df* = 5, *p* < 0.001; TER: *W* = 0.889, *Chi-sqr.* = 65.643, *df* = 5, *p* < 0.001). The highest rate of violation of the assumption was identified in the case of the metric WER (*G-G Epsilon* = 0.912), followed by PER (*G-G Epsilon* = 0.919), on the contrary, the lowest for the metric TER (*G-G Epsilon* = 0.923). Overall, the rate of violation of the assumption of sphericity of the covariance matrix was low for all applied metrics, we used adjusted significance tests (WER, PER, and TER: *G-G Epsilon* < 0.923, *G-G Adj. p* < 0.001) and subsequently, we compared them with unadjusted univariate tests for repeated measure (*F* > 211.214, *p* < 0.001).

Based on the results, we reject the global H0 at the 0.001 significance level in the case of all metrics, which claims that there is no statistically significant difference in the quality of MT when translating from German to Slovak, represented by the error rate metrics PER, WER, and TER, among GT_SMT, GT_NMT, mt@ec_SMT, and eTranslation_NMT. NMTs were of statistically significantly better quality than SMTs regardless of which MT tool was used (Tables 5, 6). NMT produced by GT tool (Tables 5, 6) achieved statistically significant the lowest error rate (*PER* = 0.495, *WER* = 0.609, *TER* = 0.607). On the other hand, SMT produced by mt@ec, a EC tool (Tables 5, 6) achieved statistically significant the highest error rate (*PER* = 0.720, *WER* = 0.821, *TER* = 0.820).

**Table 5:** Bonferroni (adjustment) post-hoc test for multiple comparisons of (a) the PER and (b) WER metrics between different MT systems (GT tools or EC tools) and approaches (statistical or neural) in the German–Slovak language pair.

(a) English = 0	Mean	1	2	3	4	(b) English = 0	Mean	1	2	3	4
PER_GT_NMT	0.495	****				WER_GT_NMT	0.609	****			
PER_EC_NMT	0.548		****			WER_EC_NMT	0.664		****		
PER_GT_SMT	0.649			****		WER_GT_SMT	0.765			****	
PER_EC_SMT	0.720				****	WER_EC_SMT	0.821				****

****Homogenous groups *p* > 0.05.

**Table 6:** Bonferroni (adjustment) post-hoc test for multiple comparisons of TER metrics between different MT systems (GT tools or EC tools) and approaches (statistical or neural) in the German–Slovak language pair.

English = 0	Mean	1	2	3	4
TER_GT_NMT	0.607	****			
TER_EC_NMT	0.662		****		
TER_GT_SMT	0.763			****	
TER_EC_SMT	0.820				****

****Homogenous groups *p* > 0.05.

We conclude that the assumption regarding better NMT quality compared to SMT has been confirmed, regardless of the language pair. We showed statistically significant differences between SMT and NMT in favor of NMT based on all metrics of error rate (WER, PER, and TER), regardless of MT tool used (Google Translate tool or the European Commission’s MT tool).

These findings indicate that the error rate in the examined texts is probably related to recall (lexical accuracy). Considering the reference, the error rate of the examined MTs is more associated with lexical accuracy, i.e., vocabulary and word omission, than grammatical accuracy, i.e., forms and structure of words and word order. This motivated us to apply residual analysis to identify and specify in more detail MT errors that occurred in individual machine translations.

### Identification of extreme differences based on the score of error rate metrics between SMT and NMT—English-Slovak machine translations

**Figure 2:**
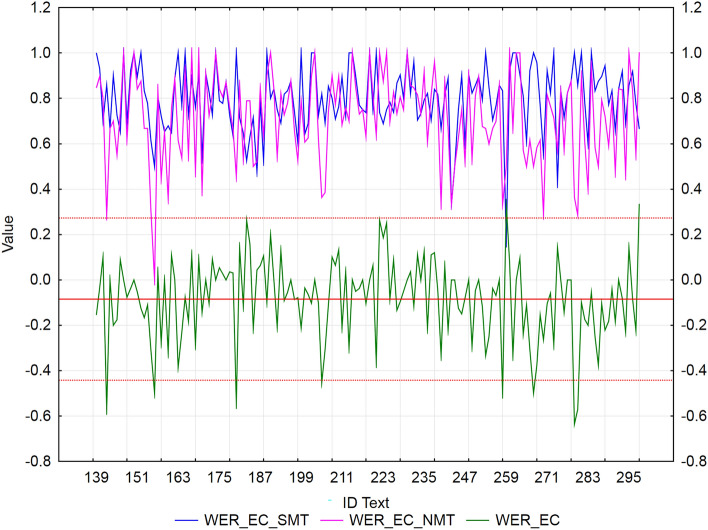
Visualization of NMT-SMT residuals for WER metric and the European Commission’s MT tool.

**Figure 3:**
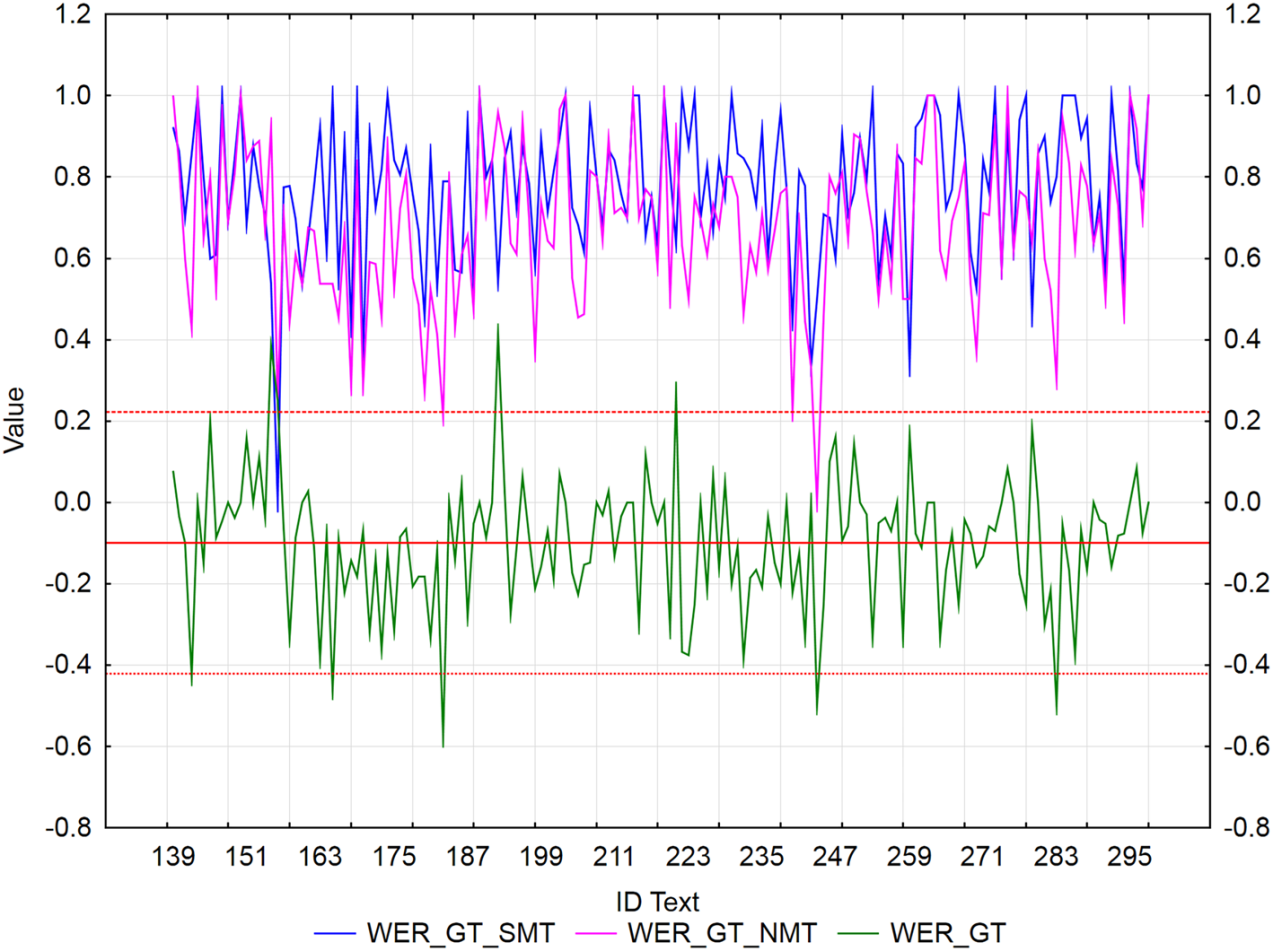
Visualization of NMT-SMT residuals for WER metric and Google translate.

**Figure 4:**
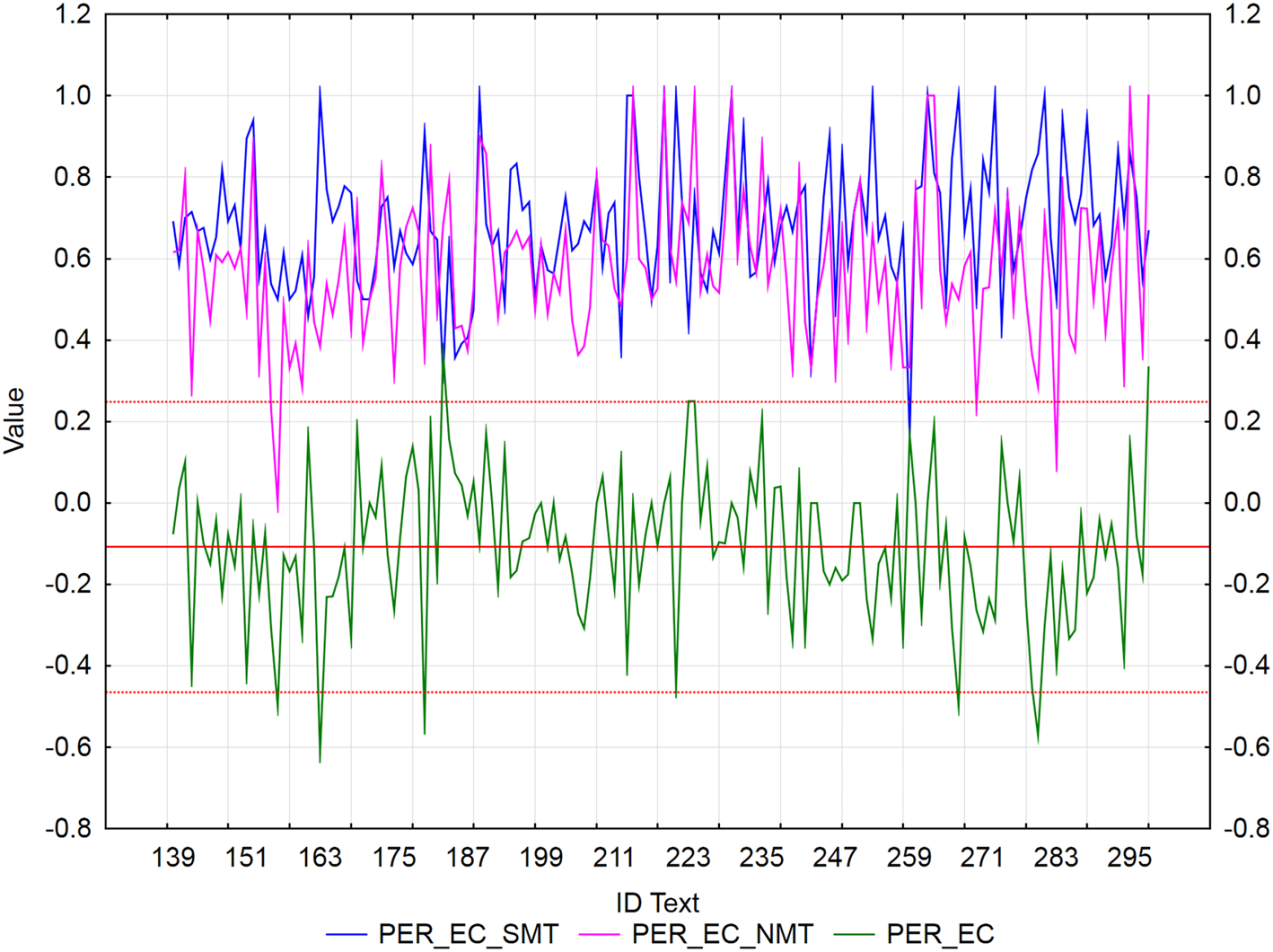
Visualization of NMT-SMT residuals for PER metric and the European Commission’s MT tool.

**Figure 5:**
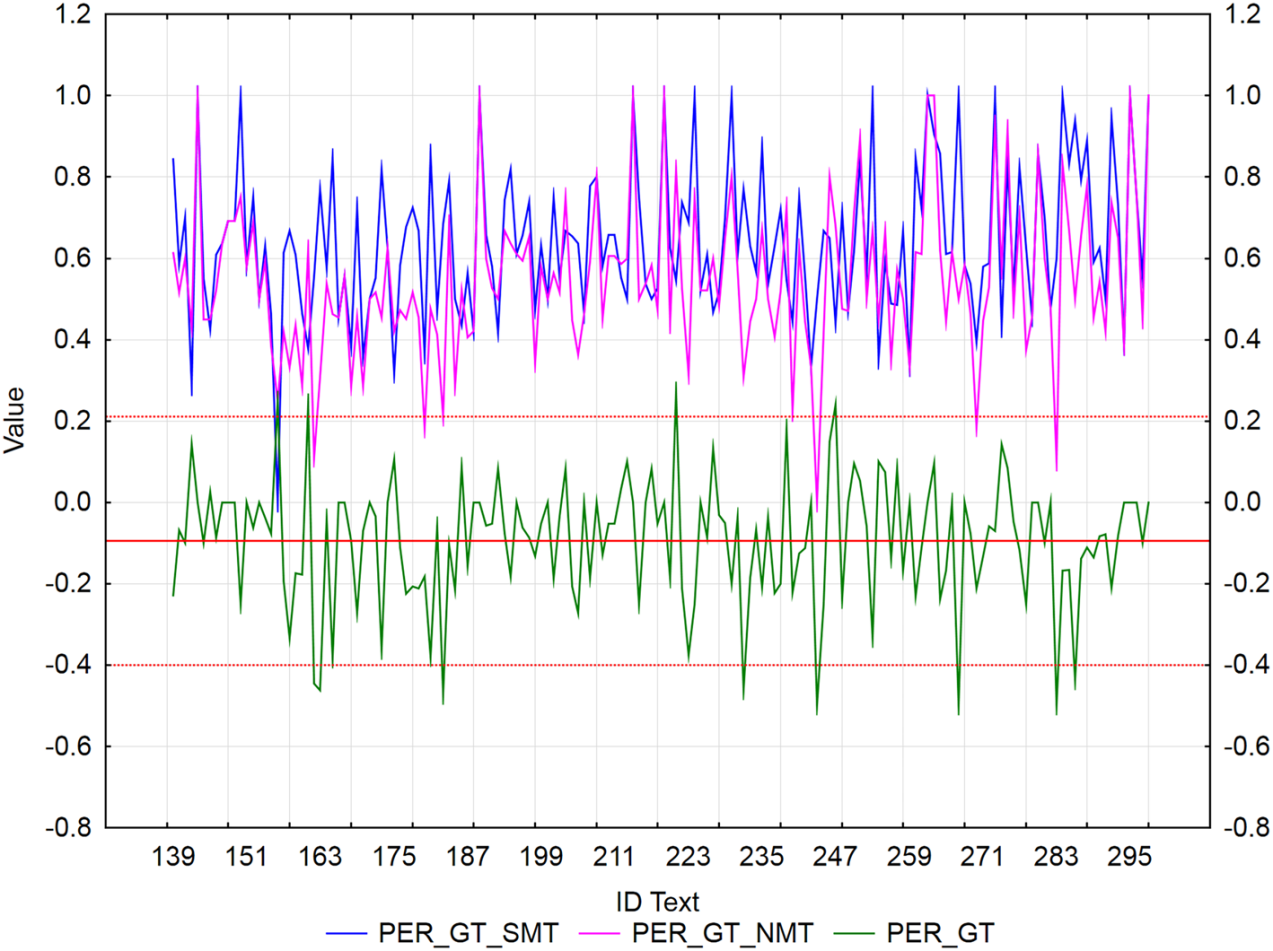
Visualization of NMT-SMT residuals for PER metric and Google translate.

**Figure 6:**
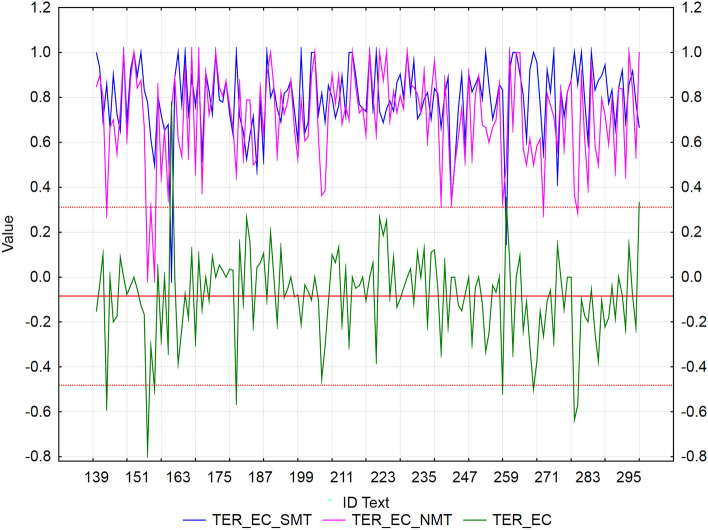
Visualization of NMT-SMT residuals for TER metric and the European Commission’s MT tool.

**Figure 7:**
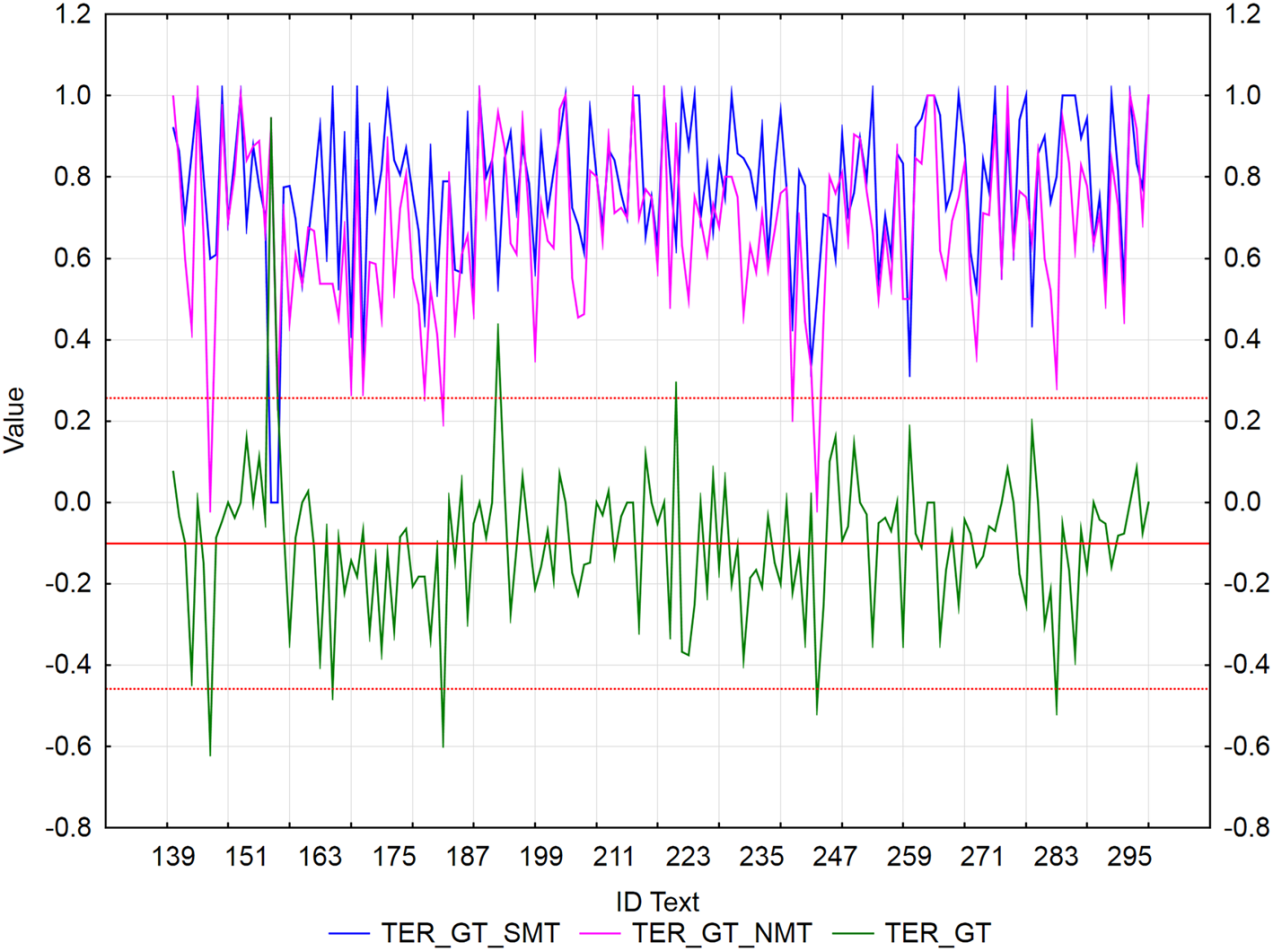
Visualization of NMT-SMT residuals for TER metric and Google translate.

We used residuals to identify texts with extreme values of error rate metrics (WER, PER, and TER) between SMT and NMT for each MT tool separately. We applied the rule ± *2sigma*, i.e., values outside the interval are considered extremes. The mean of NMT—SMT differences for all metric values (WER/PER/TER) is negative (Figs. 2, 3, 4, 5, 6, 7), which confirms our finding (previous subsection) that in terms of error rate, NMT achieved a statistically significantly lower error rate, i.e., better translation quality. The neural MT outputs were more similar to the references than the statistical MT outputs.

In the case of the European Commission’s MT tool (Fig. 2), we identified 8 texts (ID_142, ID_156, ID_180, ID_205, ID_258, ID_267, ID_279, and ID_280), that showed a statistically significantly better WER score of NMT against SMT (*residuals ≈ *− 0.5). Only 2 texts (ID_259 and ID_298) achieved a significantly better WER score of SMT against NMT (*residuals ≈ *0.33), but both texts consist of short sentences (less than 7 words, including articles), which could have had an impact on the results.

In the case of Google translate (Fig. 3), we identified 5 texts (ID_142, ID_156, ID_180, ID_205, ID_258, ID_267, ID_279, and ID_280), that showed a statistically significantly better WER score of NMT against SMT (*residuals ≈* -0.5) and 4 texts (ID_155, ID_156, ID_192, and ID_221) with a significantly better WER score of SMT against NMT (*residuals ≈ *0.35). These texts were more similar to the reference than NMT (NMT was correct, but used synonyms, which could have had an impact on the results).

In the case of the European Commission’s MT tool (Fig. 4), we identified 5 texts (ID_156, ID_163, ID_180, ID_267, and ID_280), that showed a statistically significantly better PER score of NMT against SMT (*residuals ≈* -0.55). Only 4 texts (ID_183, ID_223, ID_224, and ID_298) achieved a significantly better PER score of SMT against NMT (*residuals ≈ *0.3). Again, they were texts with short sentences, and NMT added extra words compared to the reference, which could have had an impact on the results.

In the case of Google translate (Fig. 5), we identified 8 texts (ID_162, ID_163, ID_183, ID_232, ID_244, ID_267, ID_283, and ID_286), that showed a statistically significantly better PER score of NMT against SMT (*residuals ≈* -0.45) and 4 texts (ID_156, ID_161, ID_221, and ID_247) with a significantly better PER score of SMT against NMT (*residuals ≈* 0.25). These texts were more similar to the reference than NMT.

In the case of the European Commission’s MT tool (Fig. 6), we identified 8 texts (ID_142, ID_154, ID_156, ID_180, ID_258, ID_267, ID_297, and ID_280), that showed a statistically significantly better TER score of NMT against SMT (*residuals ≈ *− 0.6). Only 3 texts (ID_161, ID_259, and ID_298) achieved a significantly better TER score of SMT against NMT (*residuals ≈ *0.33).

In the case of Google translate (Fig. 7), we identified 5 texts (ID_145, ID_165, ID_183, ID_244, and ID_283), that showed a statistically significantly better TER score of NMT against SMT (*residuals ≈ *− 0.5) and 3 texts (ID_155, ID_192, and ID_221) with a significantly better score of SMT against NMT (*residuals ≈ *0.35). These texts were more similar to the reference than NMT.

Based on our results, we can infer that the issue in MT systems lies in lexical semantics rather than in word order in the case of neural machine translation.

## Discussion

The applied automatic metrics are based on a comparison with a reference, which, in our case, was created independently (pure human translation, not affected by MT output). This could cause a distortion of MT quality, but it did not affect the comparison of SMT and NMT because we used the same reference in both cases.

Based on corpus statistics (Table 1), we assumed that NMT outperforms SMT with respect to the lexico-grammatical features of the examined texts (frequency of nouns, adjectives, and verbs).

Based on analysis results, we can conclude that NMT demonstrated higher quality than SMT in terms of error rate. All automatic metrics achieved lower scores for neural MT compared to statistical MT, i.e., NMT outperformed SMT. The most serious issues of SMT include a shift in part-of-speech, omission or addition of words, and inflection. The word order was not such a serious issue for neural MT, which we explain by the fact that it was a translation into Slovak, which has a loose word order, unlike English, which has a strict word order (SWO).

Regarding the accuracy of the strings (represented by metrics WER and TER), SMT produced approximately the same error rate, whether it was SMT produced by GT or produced by mt@ec, EC tool (Tables 2, 5b), which is noteworthy. Both MT tools performed at a very similar level. On the contrary, due to the similarity of the strings (represented by metric PER), NMT produced approximately the same error rate, whether it was NMT produced by GT or produced by mt@ec, EC tool (Tables 3, 5a). We explain this fact by the character of the examined texts. They were of the journalistic style (newspaper writing) with no specific vocabulary or complex syntax, so MT tools did not require training on a specific text-domain. SMT showed similar error rates whether it was trained on a general text-domain (GT) or a specialized text-domain, such as administrative texts (EC). In the case of the metric PER, which only focuses on word similarity (independent of word position) and does not take into account word order and extra words, NMT achieved approximately the same error rate, whether it was NMT produced by GT or produced by mt@ec (Tables 3, 5b).

To validate the obtained results, we employed automatic metrics such as BLEU, COMET, and CharacTER to ensure the reliability of error rate metrics for both language directions (BLEU: *G-G Epsilon* < 0.940, *G-G Adj. p* < 0.001; COMET: *G-G Epsilon* = 0.812, *G-G Adj. p* < 0.001, and characTER: *G-G Epsilon* = 0.932, *G-G Adj. p* < 0.001). The results (Table 7) nearly fully correspond with the results for the metrics PER, WER, and TER for English-Slovak machine translation (Tables 2, 3, 4) as well as German-Slovak machine translation (Tables 5, 6). NMTs were of statistically significantly better quality than SMTs regardless of which MT tool and language direction were used (Table 7). NMT produced by GT (Table 7) achieved statistically significantly the lowest error rate (*CharacTER* = 0.481) and statistically significantly the highest accuracy (*COMET* = 0.887, *BLEU_1* = 0.514, *BLEU_2* = 0.227, *BLEU_3* = 0.164, *BLEU_4* = 0.097). On the other hand, SMT produced by mt@ec, EC tool (Table 7) achieved statistically significantly the highest error rate (*CharacTER* = 0.688) and statistically significantly the lowest accuracy (*COMET* = 0.662, *BLEU_1* = 0.303, *BLEU_2* = 0.115, *BLEU_3* = 0.044). According to the metric BLEU_4 (Table 7f), both SMT systems (mt@ec and GT) form one homogeneous group, i.e., they achieved the same lowest quality (*p* > 0.05).

**Table 7:** Bonferroni (adjustment) post-hoc test for multiple comparisons of (a) the CharacTER, (b) COMET, (c) BLEU_1, (d) BLEU_2, (e) BLEU_3, and (f) BLEU_4 metrics between different MT systems (GT tools or EC tools) and approaches (statistical and neural).

(a) All groups	Mean	1	2	3	4	(b) All groups	Mean	1	2	3	4
CharacTER_GT_NMT	0.481	****				COMET_EC_SMT	0.662	****			
CharacTER_EC_NMT	0.544		****			COMET_GT_SMT	0.739		****		
CharacTER_GT_SMT	0.594			****		COMET_EC_NMT	0.857			****	
CharacTER_EC_SMT	0.688				****	COMET_GT_NMT	0.887				****

(c) All groups	Mean	1	2	3	4	(d) All groups	Mean	1	2	3	4
BLEU_1_EC_SMT	0.303	****				BLEU_2_EC_SMT	0.115	****			
BLEU_1_GT_SMT	0.382		****			BLEU_2_GT_SMT	0.153		****		
BLEU_1_EC_NMT	0.463			****		BLEU_2_EC_NMT	0.220			****	
BLEU_1_GT_NMT	0.514				****	BLEU_2_GT_NMT	0.277				****

(e) All groups	Mean	1	2	3	4	(f) All groups	Mean	1	2	3	
BLEU_3_EC_SMT	0.044	****				BLEU_4_EC_SMT	0.017	****			
BLEU_3_GT_SMT	0.072		****			BLEU_4_GT_SMT	0.032	****			
BLEU_3_EC_NMT	0.119			****		BLEU_4_EC_NMT	0.062		****		
BLEU_3_GT_SMT	0.164				****	BLEU_4_GT_NMT	0.097			****	

****Homogenous groups *p* > 0.05.

To analyze the relationships between the automatic metrics of error rate (PER, WER, and TER) and the metrics we chose as a baseline—valid criteria (BLEU_1-4, CharacTER, and COMET), we employed non-parametric correlations. Due to deviations from the normality of the automatic metrics (PER, WER, TER, BLEU_1-4, CharacTER, and COMET), we applied non-parametric Spearman rank order correlations to both language directions (*W* < 0.993, *p* < 0.001), but separately for statistical MT (Table 8) and for neural MT (Table 9).

**Table 8:** Non-parametric correlations for SMT: (a) PER × valid criterion (BLEU_1-4, CharacTER, COMET), (b) WER × valid criterion (BLEU_1-4, CharacTER, COMET), (c) TER × valid criterion (BLEU_1-4, CharacTER, COMET).

(a) PER_GT_SMT				PER_EC_SMT			
GT_SMT	R	t(N − 2)	*p* value	EC_SMT	R	t(N − 2)	*p* value
BLEU_1	− 0.92	− 65.107	< 0.001	BLEU_1	− 0.93	− 66.824	< 0.001
BLEU_2	− 0.75	− 30.742	< 0.001	BLEU_2	− 0.74	− 29.891	< 0.001
BLUE_3	− 0.58	− 19.152	< 0.001	BLUE_3	− 0.55	− 17.712	< 0.001
BLEU_4	− 0.39	− 11.369	< 0.001	BLEU_4	− 0.35	− 10.164	< 0.001
CharacTER	0.49	15.083	< 0.001	CharacTER	0.48	14.793	< 0.001
COMET	− 0.37	− 10.659	< 0.001	COMET	− 0.34	− 9.720	< 0.001

(b) WER_GT_SMT				WER_EC_SMT			
GT_SMT	R	t(N − 2)	*p* value	EC_SMT	R	t(N − 2)	*p *value
BLEU_1	− 0.73	− 28.521	< 0.001	BLEU_1	− 0.71	− 27.205	< 0.001
BLEU_2	− 0.67	− 24.155	< 0.001	BLEU_2	− 0.65	− 22.877	< 0.001
BLUE_3	− 0.54	− 17.097	< 0.001	BLUE_3	− 0.50	− 15.345	< 0.001
BLEU_4	− 0.36	− 10.441	< 0.001	BLEU_4	− 0.33	− 9.312	< 0.001
CharacTER	0.60	20.005	< 0.001	CharacTER	0.52	16.469	< 0.001
COMET	− 0.35	− 10.124	< 0.001	COMET	− 0.30	− 8.100	< 0.001

(c) TER_GT_SMT				TER_EC_SMT			
GT_SMT	R	t(N − 2)	*p* value	EC_SMT	R	t(N − 2)	*p* value
BLEU_1	− 0.73	− 28.720	< 0.001	BLEU_1	− 0.71	− 27.214	< 0.001
BLEU_2	− 0.67	− 24.288	< 0.001	BLEU_2	− 0.65	− 22.886	< 0.001
BLUE_3	− 0.54	− 17.116	< 0.001	BLUE_3	− 0.50	− 15.355	< 0.001
BLEU_4	− 0.36	− 10.402	< 0.001	BLEU_4	− 0.33	− 9.354	< 0.001
CharacTER	0.59	19.801	< 0.001	CharacTER	0.52	16.480	< 0.001
COMET	− 0.35	− 10.157	< 0.001	COMET	− 0.30	− 8.095	< 0.001

**Table 9:** Non-parametric correlations for NMT: (a) PER × valid criterion (BLEU_1-4, CharacTER, COMET), (b) WER × valid criterion (BLEU_1-4, CharacTER, COMET), (c) TER × valid criterion (BLEU_1-4, CharacTER, COMET).

(a) PER_GT_NMT				PER_EC_NMT			
GT_SMT	R	t(N − 2)	*p* value	EC_SMT	R	t(N − 2)	*p* value
BLEU_1	− 0.96	− 91.080	< 0.001	BLEU_1	− 0.95	− 81.806	< 0.001
BLEU_2	− 0.85	− 43.190	< 0.001	BLEU_2	− 0.78	− 33.459	< 0.001
BLUE_3	− 0.72	− 27.440	< 0.001	BLUE_3	− 0.67	− 24.399	< 0.001
BLEU_4	− 0.57	− 18.339	< 0.001	BLEU_4	− 0.48	− 14.652	< 0.001
CharacTER	0.68	24.687	< 0.001	CharacTER	0.62	20.922	< 0.001
COMET	− 0.51	− 15.980	< 0.001	COMET	− 0.49	− 14.986	< 0.001

(b) WER_GT_NMT				WER_EC_NMT			
GT_SMT	R	t(N − 2)	*p* value	EC_SMT	R	t(N − 2)	*p* value
BLEU_1	− 0.79	− 34.741	< 0.001	BLEU_1	− 0.76	− 30.891	< 0.001
BLEU_2	− 0.80	− 35.190	< 0.001	BLEU_2	− 0.70	− 26.229	< 0.001
BLUE_3	− 0.69	− 25.411	< 0.001	BLUE_3	− 0.62	− 20.887	< 0.001
BLEU_4	− 0.56	− 18.204	< 0.001	BLEU_4	− 0.47	− 14.101	< 0.001
CharacTER	0.79	34.311	< 0.001	CharacTER	0.75	30.335	< 0.001
COMET	− 0.48	− 14.494	< 0.001	COMET	− 0.44	− 12.941	< 0.001

(c) TER_GT_NMT				TER_EC_NMT			
GT_SMT	R	t(N − 2)	*p* value	EC_SMT	R	t(N − 2)	*p* value
BLEU_1	− 0.79	− 34.716	< 0.001	BLEU_1	− 0.75	− 30.828	< 0.001
BLEU_2	− 0.79	− 34.597	< 0.001	BLEU_2	− 0.70	− 26.140	< 0.001
BLUE_3	− 0.68	− 24.863	< 0.001	BLUE_3	− 0.61	− 20.624	< 0.001
BLEU_4	− 0.55	− 17.835	< 0.001	BLEU_4	− 0.46	− 13.881	< 0.001
CharacTER	0.78	33.488	< 0.001	CharacTER	0.75	30.019	< 0.001
COMET	− 0.47	− 14.446	< 0.001	COMET	− 0.43	− 12.902	< 0.001

In the case of SMT (Table 8), similar results were achieved for both MT systems (GT and EC). The examined metrics of error rate (PER, WER, and TER) positively correlate with the CharacTER metric (Table 8), indicating a moderate (> 0.3) to high (> 0.5) degree of statistically significant direct proportional dependency (*p* < 0.001). On the contrary, in the case of the metrics of accuracy (BLEU_1-4 and COMET), a negative correlation was identified (Table 8), revealing a moderate (< − 0.3) degree of dependency between the automatic metrics (PER, WER, and TER) and the metric COMET/BLEU_4. A high (< − 0.5) to very high (< − 0.7) degree of statistically significant inverse-related dependency was observed between them and the metrics BLEU_1-3 (*p* < 0.001).

Similar results were achieved in the case of NMT (Table 9). The automatic error rate metrics (PER, WER, and TER) positively correlate with the characTER error rate metric (Table 9), showing a high (> 0.5) to very high (> 0.7) degree of statistically significant direct proportional dependency (*p* < 0.001). On the contrary, in the case of the metrics of accuracy (BLEU_1-4 and COMET), a negative correlation was identified (Table 9). Between the automatic metrics (PER, WER, and TER) and the metric COMET/ BLEU_4 a moderate (< − 0.3) to a high (< − 0.5) degree of dependency was observed, and between the metrics BLEU_1-3 and automatic metrics (PER, WER, and TER), a high (< − 0.5) to very high (< − 0.7) degree of statistically significant inverse-related dependency was found (*p* < 0.001).

In the case of NMT (Table 9), higher dependencies were identified compared to SMT (Table 8), but in both cases, they reached at least a medium level of statistically significant dependency.

These results motivated us to conduct a manual error analysis for both SMT and NMT. We restricted the analysis to only 5 MT texts produced by GT tools (SMT_GT vs NMT_GT) due to its labour- and time-intensive nature. We divided the occurred errors into the following 4 categories that cover the text complexity of inflectional languages^55^: (1) predication, (2) syntactic-semantic correlativeness, (3) compound/complex sentences, and (4) lexical semantics.

SMT produced 184 errors in the category of predication, 279 errors in syntactic-semantic correlativeness, 76 errors in compound/complex sentences, and 370 errors in the category of lexical semantics. The results obtained for NMT were significantly different. In the sphere of predication 27 errors were identified, in syntactic-semantic correlativeness 106 errors, in compound/complex sentences 12 errors, and in the sphere of lexical semantics 442 errors were identified.

Our results correspond with the findings of similar studies^56,57^ which showed that SMT is more accurate in meaning (lexical accuracy), but less fluent in grammar (grammatical accuracy), and vice versa, NMT is grammatically more fluent, but less accurate in meaning (lexical semantics).

Using residual analysis, we can reveal which errors persist and, conversely, which have been eliminated or have arisen.

In the case of the European Commission’s DGT tools, when we compared SMT and NMT based on the WER metric, which takes into account not only lexical accuracy, but also grammatical correctness and word order, we found that errors most often occurred within the lexical semantics, either in (1) part of speech transformation, e.g. a noun becomes an adjective after translation with a shift in meaning, or in (2) a shift of gender, most often from masculine to feminine, or in (3) omission of commas.

Example,

- SS: The other is *the opposite*: adaptable, empathetic, flexible. (noun).
- SMT: Druhou je *opačná*. prispôsobiteľné empatický pružný. (adjective)
- NMT: Druhý je *opak*: Prispôsobivý, empatický, flexibilný. (noun)
- HT: Druhý je presným *opakom*: prispôsobivý, empatický, flexibilný. (noun)

Another (4) frequent issue was word omission and word order, e.g.

- SS: Among their number were Belgian students, French schoolchildren and British lawyers.
- SMT: Spomedzi nich boli belgické, francúzske a britské študentov, advokátov. (omission of word *students* or *schoolchildren*)
- NMT: Medzi ich počet boli belgickí *študenti*, francúzski *žiaci* a britskí právnici.
- HT: Nachádzajú sa medzi nimi belgickí *študenti*, francúzski školáci a britskí právnici.

We achieved similar results for Google Translate, but we also identified four texts in which SMT achieved a better WER score than NMT. However, after a deeper analysis we found that it was caused by using synonyms (different words with the same meaning) or by expanding the information with respect to the reference, e.g.

- SS: That equates to 5am GMT back in the *United Kingdom*.
- SMT: To sa rovná 5 hodín ráno GMT vzadu v *Spojenom kráľovstve*. (United Kingdom)
- NMT: To predstavuje vo *Veľkej Británii* 5:00 GMT. (Great Britain)
- HT: To je presne 5:00 ráno západoeurópskeho času v *Spojenom kráľovstve* (GMT).

Our findings are in line with the other studies^58–63^ that focused on comparing SMT and NMT quality across various text genres. Benkova et al.^63^ conducted similar research, using residual analysis and the automatic metric BLEU-n to compare quality between neural and statistical MT systems. They came to the conclusion that neural MT is more accurate or closer to reference than statistical MT in the translation of journalistic texts from English into Slovak. However, their focus was solely on the standard automatic metric of accuracy (BLEU), which does not always correlate with human evaluation in the case of machine translation into inflectional languages.

Our study provides new insights into the evaluation of MT quality from English and German into Slovak through automatic evaluation metrics of error rate and residuals. Residuals, combined with automatic metrics of error rate, represent, and/or indicate a new approach to quality evaluation and comparison between statistical and neural machine translation. To our knowledge, no study has applied residuals to identify extreme differences in the error rate of SMT and NMT. This approach is universal, independent of the languages, text-domains, or the MT tools used, which makes it original. Moreover, the issue related to reference translation is removed, and/or eliminated, as it is only a parameter when comparing two MT outputs.

The study has certain limitations, which are mainly related to the size of the dataset. We plan to expand our corpus size with more texts of the newspaper writing style, as well as of other styles.

## Conclusions

In our study, we demonstrated that through automatic evaluation metrics, neural machine translation achieved a lower error rate than statistical machine translation, regardless of the MT tool used. The manual error analysis of the selected smaller sub-corpus indicates that in the category of prediction (consisting of predicative categories, non-finite verb or other word class instead of finite verb functioning as a predicate, missing verb in predication, sentence with or without subject, sentence with or without agent, descriptive and reflexive passive verb forms, incorrectly identified subject in the sentence, incorrectly identified predicate in a sentence, incorrect form of a complex verb phrase, and others) and syntactic-semantic correlativeness (consisting of nominal morphosyntax, pronominal morphosyntax, numeral morphosyntax, verbal morphosyntax, word order, and other morphosyntactic phenomena), SMT showed a significantly higher error rate than NMT. Conversely, in the category of lexical semantics (adequate transfer of the words’ meaning, polysemy, homonymy, semantic and stylistic compatibility, derivation, omission, literal translation, explication, and other), NMT showed a significantly higher error rate than SMT.

Remarkably, the research also revealed considerable diversity in translation quality. As mentioned in the methodology, the MT outputs were post-edited by professional translators. We assumed that human translations and post-edited MT outputs would be at least 80% similar, therefore, we included a calculation of the text similarity through the cosine similarity into our analysis. We found that in the case of SMT, PEMT_SMT and HT there is only about 50% similarity, and in the case of NMT, PEMT_NMT and HT, there is only about 54% similarity, as expected since these were two different translation techniques (post-editing of MT output and human translation). However, in the case of post-editing, a much higher agreement and/or text similarity was assumed, which was not confirmed. In the case of PEMT_SMT and PEMT_NMT, only about 61% text similarity was observed. Even the text similarity between two post-edited MT outputs was not high; it achieved only 75% in the case of post-editing of NMT produced by GT. However, the NMT error rate dropped from about 69% to about 58% when the post-edited SMT was used as a reference.

Relying solely on the reference when determining MT quality turns out to be insufficient, but in combination with residuals, it provides more reliable results, and/or a more objective view of MT quality and the comparison of SMT and NMT.

A significant contribution of residual analysis is the identification of specific segments, in our case short texts, in which neural MT achieved a significantly lower error rate, but mainly in the identification of segments in which, on the contrary, statistical MT achieved better results, regardless of MT systems and language directions, with a focus on machine translation into inflectional and low-resourced Slovak.

## Data Availability

The dataset analysed during the current study is available in the Mendely Data repository under 10.17632/yrft7c64z6.1.
